# Sensitivity Analysis of Biosensors Based on a Dielectric-Modulated L-Shaped Gate Field-Effect Transistor

**DOI:** 10.3390/mi12010019

**Published:** 2020-12-27

**Authors:** Chen Chong, Hongxia Liu, Shulong Wang, Shupeng Chen, Haiwu Xie

**Affiliations:** Key Laboratory for Wide-Band Gap Semiconductor Materials and Devices of Education, The School of Microelectronics, Xidian University, Xi’an 710071, China; 18829029042@163.com (C.C.); chenshupeng999@126.com (S.C.); xiehaiwu.love@163.com (H.X.)

**Keywords:** dielectric-modulated L-shaped tunneling field-effect transistor (DM-LTFET), biosensor, sensitivity

## Abstract

Label-free biomolecular sensors have been widely studied due to their simple operation. L-shaped tunneling field-effect transistors (LTFETs) are used in biosensors due to their low subthreshold swing, off-state current, and power consumption. In a dielectric-modulated LTFET (DM-LTFET), a cavity is trenched under the gate electrode in the vertical direction and filled with biomolecules to realize the function of the sensor. A 2D simulator was utilized to study the sensitivity of a DM-LTFET sensor. The simulation results show that the current sensitivity of the proposed structure could be as high as 2321, the threshold voltage sensitivity could reach 0.4, and the subthreshold swing sensitivity could reach 0.7. This shows that the DM-LTFET sensor is suitable for a high-sensitivity, low-power-consumption sensor field.

## 1. Introduction

In recent years, field-effect transistor (FET) biosensors have been studied by many researchers [1,2,3,4,5]. However, metal oxide semiconductor field-effect transistors (MOSFETs) cannot break through the 60 mV/dec limit due to the conduction mechanism of thermionic emission. Tunneling field-effect transistors (TFET) can make the sub-threshold swing lower than 60 mV/dec due to its band-to-band tunneling (BTBT) conduction mechanism; therefore, TFET-based sensors are increasingly attracting researchers’ attention [6,7,8,9,10].

Dielectric modulation is used to engrave a part of the gate oxide under the gate electrode to form a nanocavity, which is then filled with biomolecules. The dielectric constant of the cavity changes (different biomolecules have different dielectric constants) and the electrical characteristics of the device also change, which is reflected in the changes in the transfer curve and sensitivity. Due to its low cost and easy operation, dielectric modulation is applied in biosensors [11,12,13,14]. Therefore, sensors made using dielectric modulation based on a TFET have been studied by many scholars [15,16,17,18]. In 2016, Kanungo et al. studied the influence of silicon germanium (SiGe) sources and n+-pocket-doped channels on dielectric modulation sensors. Studies have shown that in order to maximize the sensitivity, the proportion of germanium should be kept at 10% [19]. In 2019, through Technology Computer-Aided Design (TCAD) simulations that were used to identify the sensitivity of a double-gate dielectric modulation junctionless TFET for biomolecule recognition, Wadhwa and Raj studied the influence of the cavity length, different biomolecules, and different charges on the drain current, subthreshold swing (*SS*), and *I_on_*/*I_off_* [20]. Mohammad et al. conducted research on biosensors based on a SiGe source dual-gate TFET. The effects of cavity length, the presence or absence of biomolecules, and biomolecules with different charge concentrations on the sensitivity of the sensor were studied. However, the study did not carefully consider the influence of different biomolecules or the positive charge of the biomolecules on the sensor sensitivity [21]. In [22], Wadhwa studied the effect of the fill factor of biomolecules on the transfer characteristics of a dual-gate junctionless TFET sensor.

However, most of the studies in the literature are based on dual-gate TFET sensors, and there are few studies on single-gate sensors. Dual-gate TFETs need cavities to be etched under both gate electrodes. In a dielectric-modulated L-shaped tunneling field-effect transistor (DM-LTFET), only one cavity needs to be trenched under the gate electrode, which is then filled with biomolecules; this setup is simple to operate and low in cost. Because the source and gate overlap, the tunneling area of an LTFET is much larger than that of a planar TFET. The greater the on-state current, the better the electrical characteristics of the LTFET. Therefore, the sensitivity of a DM-LTFET is also higher. In this study, the cavity depth was deep enough, and the gate control ability was stronger. 

In this study, the performance and underlying working mechanism of DM-LTFET-based biosensors were investigated. A detailed study was carried out to develop a comprehensive understanding of the working principle of the proposed biosensors and is presented as follows. Section 2 characterizes the basic device structure, simulation model, and method. Section 3 discusses the influence of different parameters on the sensitivity of a DM-LTFET biosensor. In detail, the influences of different biomolecules (different dielectric constants, different biomolecules), the cavity thickness and charged biomolecules on transfer characteristics, and the current sensitivity and threshold voltage sensitivity of the proposed sensor were studied. Section 4 concludes the paper.

## 2. Device Structures and Simulation Approach

**Figure 1:**
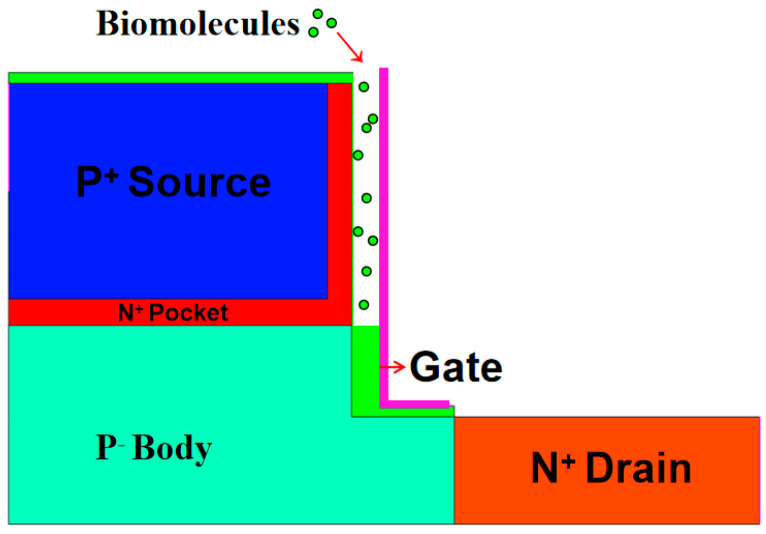
Schematic cross-sectional view of a dielectric-modulated L-shaped tunneling field-effect transistor (DM-LTFET) biosensor.

A cross-sectional view of a DM-LTFET-based biosensor is shown in Figure 1. In this simulation, the source, drain, channel, and substrate material were all silicon. HfO_2_ was used as the gate dielectric. In order to make the sensitivity parameter change more obvious, a gate metal work function that allowed for easier tunneling was adopted. This is why the metal work function *Φ_MS_* = 4.2 eV (over the HfO_2_ gate oxide) was chosen. The cavity was etched under the gate electrode and filled with small biomolecules, thereby realizing the function of a dielectric modulation biosensor. The exhaustive physical and technological parameters of the structure in the DM-LTFET are shown in Table 1. In this study, six kinds of small biomolecules with different dielectric constant values (1, 2.5, 5, 11, and 23) were used to fill nanogap cavities of different thicknesses (5, 7, 9, 11, and 13 nm) and were given different amounts of charge to be studied and analyzed when the DM-LTFET was in the on-state (*V_d_* = 0.5 V, *V_g_* = 1 V, *V_s_* = 0 V). This was mainly done by analyzing parameters such as the threshold voltage sensitivity (*S_Vth_*), current sensitivity (*S_cur_*), and sub-threshold swing sensitivity (*S_SS_*), which can respectively be expressed as [23]:

(1) $$S_{V_{th}}=\frac{V_{th(air)}-V_{th(bio)}}{V_{th(air)}}$$

(2) $$S_{cur}=\frac{I_{on(bio)}-I_{on(air)}}{I_{on(air)}}$$

(3) $$S_{SS}=\frac{SS_{air}-SS_{bio}}{SS_{air}}$$

The performance of the proposed DM-LTFET biosensor was simulated using computer-aided design (Sentaurus, O-2018.06-SP2, Sysnopsys, Mountain View, CA, USA). In order to simulate the device parameters accurately, suitable models were selected.

The magnitude of the tunneling current has a strong dependence on the degree of band bending and the boundary profile. The nonlocal tunneling model is more consistent with the actual situation of the TFET simulation. The model considered that every point of the electric field on the tunneling path was a variable, which meant that the BTBT tunneling probability depended on the band bending at the tunneling junction. Hence, the nonlocal BTBT model was adopted in this study. The rate of the BTBT tunneling is expressed as:

(4) $$G_{BTBT}=A{\left(\frac{E}{E_{0}}\right)}^{P}\exp(-\frac{B}{E_{0}})$$

where *E*_0_ = 1 V/cm, *P* = 2.5, *A* = 4 × 10^14^/cm^3^∙s and *B* = 9.9 × 10^6^ V/cm (the values of *E*_0_ and *P* are default values and the values of parameters *A* and *B* are obtained through model calibration [24]).

Because the cavity was filled with small biomolecules, it was necessary to introduce the bimolecular recombination model to calibrate the recombination model in this area. The bimolecular recombination rate is given by:

(5) $$R_{bimolec}=\gamma \cdot \frac{q}{\varepsilon_{0}\varepsilon_{r}}\cdot \left(\mu_{n}+\mu_{p}\right)\left(np-n_{i,eff}^{2}\frac{n_{se}}{n_{se}^{eq}}\right)$$

where *γ* is a prefactor for the singlet exciton; *q* is the elementary charge; *ε*_0_ and *ε_r_* denote the free space and relative permittivities, respectively; *μ_n_* and *μ_p_* are the electron mobility and hole mobility, respectively; *n* and *p* are the electron concentration and hole concentration, respectively; *n_i,eff_* is the effective intrinsic carrier concentration; *n_se_* is the singlet exciton density; $n_{se}^{eq}$ denotes the singlet-exciton equilibrium density.

## 3. Results and Discussion

This section mainly discusses the analysis and the simulation results. The sensitivity analysis of a sensor requires a certain comparative reference; therefore, this study used air, which filled the cavity, as a reference for discussion. The effects of five different biomolecules, seven different cavity thicknesses, and six different charged biomolecules on the sensitivity of the device were studied.

### 3.1. Impact of Different Biomolecules in the DM-LTFET

This section discusses the effect of filling the cavity with small biomolecules that had different dielectric constants on the sensitivity of the proposed sensor when the cavity thickness was 5 nm (*T_c_* = 5 nm).

**Figure 2:**
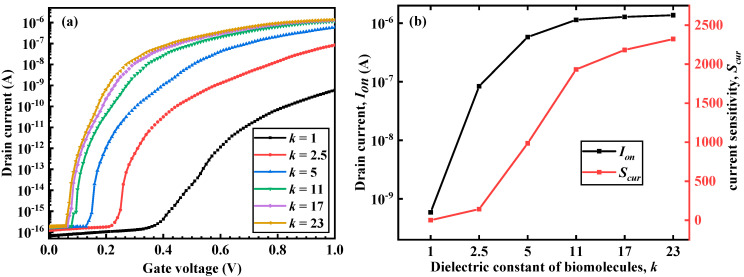

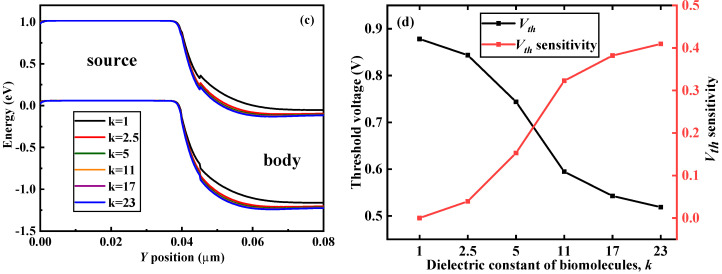
(**a**) Transfer characteristics, (**b**) *I_on_* and *S_cur_*, (**c**) energy band variation with respect to the *y*-axis, and (**d**) *S_Vth_* of the DM-LTFET biosensor for different values of *k* at *V_d_* = 0.5 V, *V_g_* = 1 V, and *T_c_* = 5 nm.

Figure 2 shows the transfer characteristic, *S_cur_*, energy band variation, and *S_Vth_* of the DM-LTFET in the on-state when different biomolecules filling the cavity provided different dielectric constants. As can be seen in Figure 2a,b, as the dielectric constant increased, the on-state current (*I_on_*) of the DM-LTFET increased, and *S_cur_* also increased. At the same time, when the dielectric constant was greater than 10, the distance between the transfer curves of the device became smaller, and the increases in *I_on_* and *S_cur_* also became smaller. Figure 2c is an energy band diagram taken along the *y*-axis along the source, pocket, and body regions. It can be seen from Figure 2c that as the dielectric constant of the biomolecules increased, the gate control capability of the DM-LTFET became stronger, and the energy band of the body region became lower. Consequently, the probability of tunneling through the source–body junction was greater, and therefore, the greater the current collected by the drain, the greater the *I_on_* and *S_cur_*. At the same time, when *k* was greater than 10, the energy band change of the body region was also very small, which was consistent with the change in the transfer curve. Figure 2d shows that as *k* increased, the threshold voltage (*V_th_*) of the DM-LTFET decreased and the DM-LTFET was easier to turn on. It can be seen from the energy band diagram of Figure 2c that as *k* increased, the more the band bent, the smaller the gate voltage required for the LTFET to reach the on state, that is, the smaller the *V_th_*. At the same time, the *S_Vth_* of the sensor also improved. Simultaneously, when *k* was greater than 11, the band bending amplitude became smaller; therefore, the *V_th_* increase was also very small when *k* was greater than 11. Therefore, when *k* was greater than 11, *S_Vth_* tended to be saturated.

**Figure 3:**
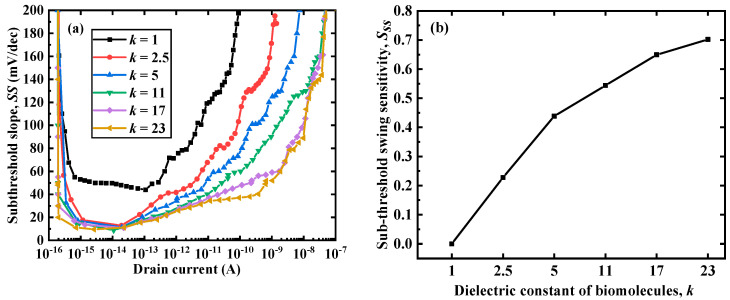
(**a**) *SS*–*I_d_* characteristics and (**b**) *S_SS_* of the DM-LTFET with different biomolecules when *V_d_* = 0.5 V, *T_c_* = 5 nm, and *V_g_* = 1 V.

The *SS* is defined as the amount of change in the gate voltage required to reduce the drain current by an order of magnitude. The *SS* is given by:

(6) $$SS=\frac{dV_{G}}{d\Psi_{S}}\frac{d\Psi_{S}}{d(\log_{10}I_{D})}≅(1+\frac{C_{d}}{C_{ox}})\ln10\frac{KT}{q}$$

where *V_G_* is the gate voltage, Ψ*_S_* is the potential, *I_D_* is the leakage current, *C_d_* is the depletion capacitance, *C_ox_* is the gate oxide layer capacitance, *K* is the Boltzmann constant, *T* is the temparature, and *q* is the charge. Figure 3 shows the *SS*–drain current characteristic curve and the change curve of *S_SS_* under different *k* values. It can be clearly seen from Figure 3a that as *k* increased, the *SS* of the device decreased. Furthermore, the larger the *k*, the larger the current range where *SS* was lower than 60 mV/dec, and the better the performance of the DM-LTFET. As *k* increased, *C_ox_* increased and *C_d_* decreased (because the width of the depletion layer decreased, thus *C_d_* decreased); therefore, *SS* increased. As depicted in Figure 3b, as *k* increased, *S_SS_* also increased. Furthermore, when *k* was greater than 10, the *S_SS_* variation range of the proposed sensor became smaller. This was due to the fact that when *k* was greater than 10, the width of the depletion layer reached the maximum, such that *C_d_* changed little but *C_ox_* still increased; therefore, the *S_SS_* change range became smaller. 

### 3.2. Impact of Different Cavity Thickness in DM-LTFET

From the results of the previous section, we know that when *k* = 23, the DM-LTFET sensor had the strongest sensitivity. Therefore, in order to study the effect of different cavity thicknesses on the proposed sensor characteristics more clearly, this section discusses the results of the DM-LTFET being studied under the condition of *k* = 23.

**Figure 4:**
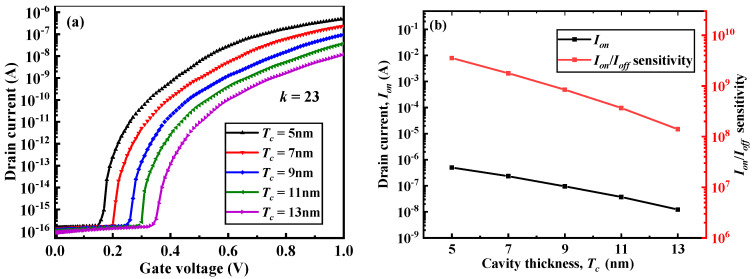
(**a**) Transfer characteristics and (**b**) *I_on_* and *I_on_*/*I_off_* sensitivity of the DM-LTFET biosensor for different values of the cavity thickness (*T_c_*) at *V_d_* = 0.5 V, *V_g_* = 1 V, and *k* = 23.

**Figure 5:**
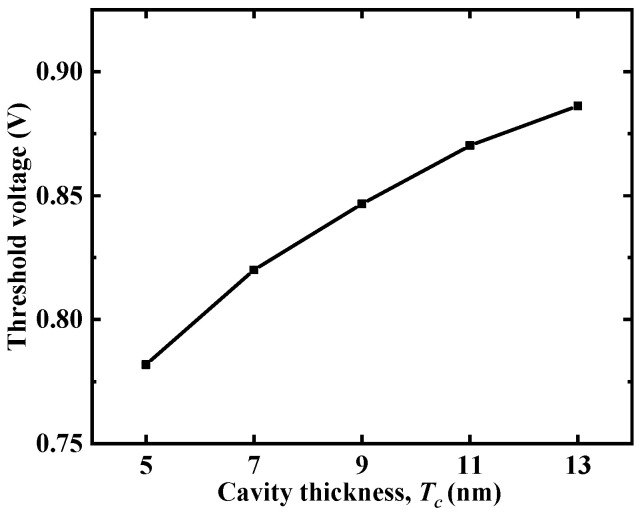
Threshold voltage of the DM-LTFET at *V_g_* = 1 V, *V_d_* = 0.5 V, and *k* = 23.

Figure 4 illustrates that with an increase in the cavity thicknesses (*T_c_*), the transfer curve of the device moved to the lower-right corner and the *I_on_* and *I_on_*/*I_off_* sensitivity of the DM-LTFET sensor became smaller. As *T_c_* increased, the actual gate oxide thickness under the gate electrode increased. When the same gate voltage was applied, the energy band of the body region of the device with a smaller *T_c_* bent more severely such that the tunneling current and the current collected by the drain electrode was larger. However, the drain current under off-state conditions did not change much. Therefore, as *T_c_* increased, the *I_on_*/*I_off_* also decreased. Figure 5 depicts that as *T_c_* increased, the device became more and more difficult to turn on, and the threshold voltage also increased. 

### 3.3. Impact of Charged Biomolecules on the DM-LTFET

The biomolecules that filled the cavity in the previous sections were uncharged; therefore, this section mainly discusses the effects of the differently charged biomolecules on the DM-LTFET sensor. In this study, the DM-LTFET biosensor detected the charged concentration of sensitive materials in the range of 10^10^–10^13^ cm^−2^, which is a wide detection range compared with other sensors [25].

**Figure 6:**
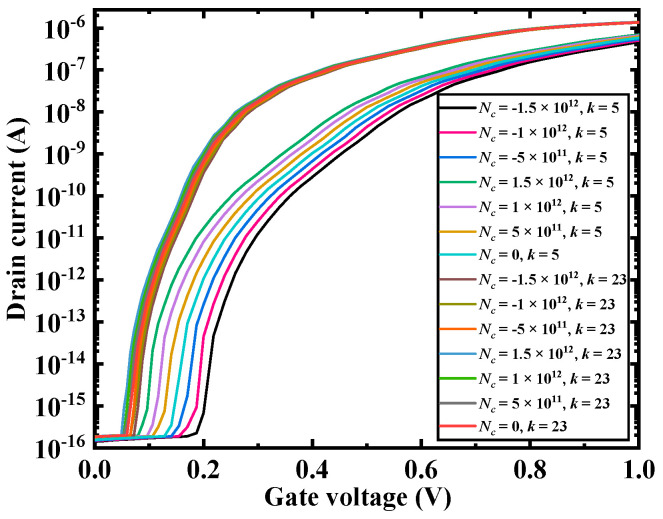
Transfer characteristics of the DM-LTFET biosensor for different dielectric constants and biomolecules charges at *V_d_* = 0.5 V, *V_g_* = 1 V, and *T_c_* = 5 nm.

Figure 6 shows the transfer curves of biomolecules with different *k* values when the charge amount was different. When the biomolecules were positively charged, the transfer curve shifted to the left as the amount of charge increased. However, when the biomolecules were negatively charged, as the amount of charge increased, the transfer curve shifted to the right. Moreover, as the value of *k* increased, the transfer curve also shifted to the left, which was consistent with the results in the previous section.

**Figure 7:**
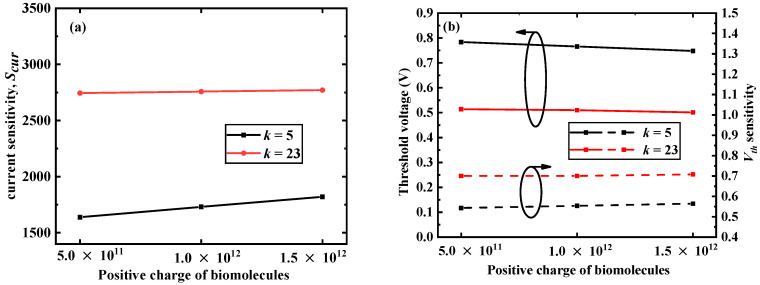
(**a**) Effect of different positive charges of the biomolecules on the current sensitivity and (**b**) *V_th_* and *S_Vth_* of the DM-LTFET at *V_g_* = 1 V, *V_d_* = 0.5 V, *k* = 2.5 and 23, and *T_c_* = 5 nm.

Figure 7 depicts the influence of different positive charges of different biomolecules on *S_cur_*, *V_th_*, and *S_Vth_*. It can be seen from Figure 7a that for a given value of *k*, with an increase in the charge of the biomolecules, *S_cur_* slowly increased. Figure 7b shows that as the amount of positive charge increased, the *V_th_* of the device slowly decreased, and *S_Vth_* slowly increased. As the amount of positive charge increased, the equivalent gate voltage applied to the gate increased, and the DM-LTFET was more likely to be turned on; therefore, *V_th_* decreased and *I_on_* increased. This further increased *S_cur_* and *S_Vth_*.

**Figure 8:**
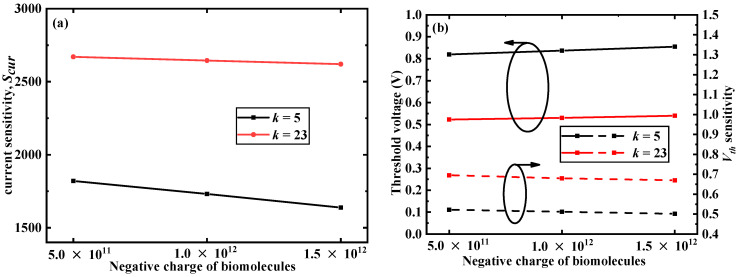
(**a**) Effect of different negative charge of biomolecules on current sensitivity and (**b**) *V_th_* and *S_Vth_* of the DM-LTFET at *V_g_* = 1 V, *V_d_* = 0.5 V, *k* = 5 and 23, and *T_c_* = 5 nm.

In Figure 8, the difference from the positively charged case was that at a given value of *k*, with more negative charges, *S_cur_* slowly decreased and *S_Vth_* also slowly decreased. At the same time, *V_th_* increased. As the amount of negative charge increased, the equivalent gate voltage applied to the gate decreased and it was harder to turn on the DM-LTFET; therefore, *V_th_* increased and *I_on_* decreased. This further decreased *S_cur_* and *S_Vth_*.

In general, as the amount of the charge of the biomolecules changed, the sensitivity of the device also changed slightly, which was much smaller than the change in sensitivity caused by changing the *k* value of the biomolecules.

### 3.4. Comparison with the Biosensor-Based TFET

**Figure 9:**
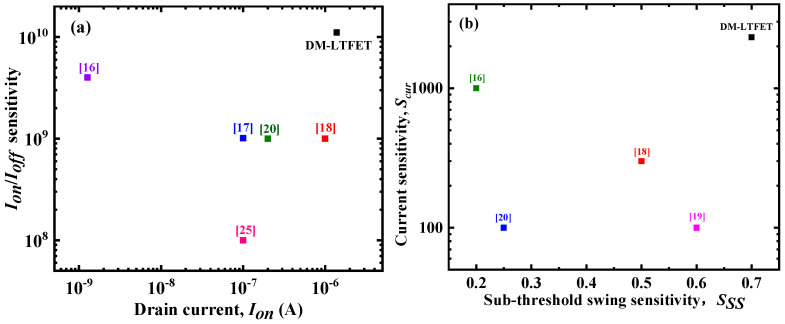
Comparison of the *I_on_*/*I_off_* sensitivity and current sensitivity of the DM-LTFET sensor with the other TFET based sensors in terms of (**a**) the on-state current and (**b**) the sub-threshold swing sensitivity.

In Figure 9a,b, we show the comparison of the DM-LTFET metrics with previously published papers. It can be clearly seen from Figure 9a that, compared with previously published papers [16,17,18,20,26] (which have double gates), the DM-LTFET could simultaneously have a larger on-state current and *I_on_*/*I_off_* sensitivity. At the same time, it can be obviously seen from Figure 9b that the current sensitivity and sub-threshold swing sensitivity of the proposed structure were higher than those of the past published papers [16,18,19,20].

## 4. Conclusions

In conclusion, the sensitivity of the DM-LTFET sensor was relatively high, which is very suitable for applications in the field of ultra-sensitive, low-consumption biosensors. The sensitivity of the DM-LTFET sensor was mainly investigated by studying the transfer curve, current sensitivity, and threshold voltage sensitivity of the proposed structure with different dielectric constants, cavity thicknesses, and charged biomolecules. It can be seen from the simulation results that the greater the relative permittivity of the biomolecules, the smaller the cavity, the greater the amount of positive charge, and the higher the sensitivity of the proposed sensor. Therefore, DM-LTFET sensors have profound development potential and market prospects.

## Figures and Tables

**Table 1:** Device parameters used for the simulation.

Parameter Name	Symbol	Value	Unit
Pocket thickness	*T_p_*	5	nm
Oxide thickness	*T_ox_*	2	nm
Channel doping	*N_c_*	1 × 10^15^	cm^−3^
Source doping	*N_s_*	1 × 10^20^	cm^−3^
Drain doping	*N_d_*	1 × 10^18^	cm^−3^
Pocket doping	*N_p_*	1 × 10^19^	cm^−3^
Source length	*L_s_*	68	nm
Drain length	*L_d_*	65	nm
Cavity height	*H_c_*	45	nm
Gate work function	*Φ_ms_*	4.2	eV
